# Stated Meeting, March 4, 1902

**Published:** 1902-04

**Authors:** F. W. McGuire

**Affiliations:** Secretary


*Stated Meeting, March 4, 1902.*

REPORTED BY F. W. McGUIRE, M. D., Secretary.

Stated meeting of the Buffalo Academy of Medicine was held at the Academy rooms, Public Library Building, Tuesday, March 4, 1902.

In the absence of the president, Dr. Charles G. Stockton, vice-president Dr. HARVEY R. GAYLORD acted as presiding officer and called the meeting to order at 9 p.m.

The minutes of the last stated meeting, held January 28, 1902, were read and approved.

The council recommended the election of Drs. David Everett Wheeler and Louis J. Beyer for resident membership. They were separately balloted for and elected fellows of the Academy.

Nomination of officers resulted as follows: For president, Drs. Ernest Wende and Herman E. Hayd; treasurer, Dr. Charles S. Jewett; trustee, Drs. Eugene Smith and Stephen Y. Howell.

A communication was received from Dr. Charles G. Stockton, expressing his regret at his enforced absence from the meeting.

It was moved and seconded that a committee of five be appointed to investigate the charges made by Dr. Julius Pohlman against St. Mary's Maternity and Orphan Asylum, the committee to report at the next stated meeting of the Academy. The chair appointed Drs. William Warren Potter, Ernest Wende, Henry R. Hopkins, Francis E. Fronczak and Thomas F. Dwyer as such committee.

The program for the evening was under the auspices of the section on surgery, and consisted of a paper by Dr. ROSWELL PARK, entitled

### THE ADVANTAGE OF EARLY SURGICAL INTERVENTION IN BORDERLAND CASES.

The paper was discussed by Drs. Herman E. Hayd, A. L. Benedict, E. E. Blaauw and Eugene Smith.

A motion was made by Dr. PARK that suitable expressions of regret be sent to the relatives of the late Dr. Moore, of Rochester. Carried. The committee appointed to draw up such resolutions were Drs. J. W. Grosvenor, Herman E. Hayd and Eugene A. Smith.

Attendance, 40. Adjourned at 11 p.m.

---

THERE are now 63 vacancies in the medical department of the army, the largest number in the history of the corps. The next examination will be held in Washington, D.C., April 7.—*Medical Age*.

